# The impact of leader mindfulness in communication on employees' psychological safety

**DOI:** 10.3389/fpsyg.2025.1540820

**Published:** 2025-08-13

**Authors:** Sijin Du, Wenli Xie

**Affiliations:** ^1^School of Economics and Management, Taizhou Polytechnic College, Taizhou City, Jiangsu Province, China; ^2^Office of Human Resources, Taizhou Polytechnic College, Taizhou City, Jiangsu Province, China; ^3^College of Finance and Economics, Qinghai University, Xining City, Qinghai Province, China; ^4^School of Marxism, Taizhou Polytechnic College, Taizhou City, Jiangsu Province, China; ^5^School of Marxism, Heilongjiang University, Harbin City, Heilongjiang Province, China

**Keywords:** leader mindfulness in communication, employees' psychological safety, leader empathy, interpersonal trust, employee mindfulness

## Abstract

**Introduction:**

This study investigated the mediating role of Leader Empathy and Interpersonal Trust between Leader Mindfulness in Communication and Employees' Psychological Safety. In addition, it also examined the positive moderating effect of employee mindfulness on multiple variables.

**Method:**

The data collection for the study was conducted in two stages, with an interval of 4 months. The data collection method is to conduct a questionnaire survey on 506 employees. The data analysis phase was conducted using Amos and SPSS statistical software.

**Results:**

The results indicate that the leader mindfulness in communication has a positive impact on employees' psychological safety. Leader empathy and interpersonal trust play a mediating role between the leader mindfulness in communication and employees' psychological safety. Employee mindfulness plays a positive moderating role among multiple variables.

**Discussion:**

Employees exhibit a very open and welcoming attitude toward the form of leader mindfulness in communication. Leaders can significantly alleviate the stress and psychological issues faced by employees through this approach. Practical strategies include promoting mindfulness training and requiring leaders to adopt leader mindfulness in communication. The study provides feasible suggestions to alleviate employees' psychological problems, enhance employees' psychological safety, create a harmonious and stable working environment, and improve organizational efficiency.

## 1 Introduction

Currently, while digital technology and artificial intelligence continue to enhance organizational efficiency, they also render challenges and threats more tangible for organizations. Particularly as incremental markets become fully partitioned and competition shifts to existing market shares, organizations must elevate their technological capabilities and deliver additional consumer services. This serves as a critical strategy for maintaining market share, yet simultaneously intensifies involutional competition among organizations (Obrenovic et al., 2020). Under such circumstances, neither organizations, leaders, nor employees remain insulated from these pressures. The challenges confronting organizations will inevitably be decomposed by leaders into concrete tasks, thereby cascading down to frontline employees. This makes employees prone to negative emotions and reduces their sense of psychological safety. Employees' psychological safety can be defined as their perception of receiving positive feedback during work and interpersonal communication in the workplace (Hebles et al., 2022). The sources of this positive feedback are multifaceted, but mainly come from familiar external environments and recognition from leaders. As a subjective feeling, this determines how employees approach their work with a certain mindset and approach. The transmission of pressure by leaders suppresses the positive feedback that employees receive during the work process. This makes them unable to work with a relaxed mindset and afraid to publicly express their opinions and suggestions, often fearing criticism, punishment, or being replaced. This will lead to employees being more cautious and passive in facing work tasks, and making corresponding defensive or confrontational behaviors, thereby posing challenges to their careers. Obviously, its decline not a good experience for both employees and organizations. For organizations, it can reduce organizational efficiency, trigger team conflicts, and create a tense atmosphere (Liu et al., 2024; Rafiei et al., 2024). For employees, it can reduce their sense of happiness, trigger depression, and lead to violent behavior (Liu et al., 2019; Jiang et al., 2023).

The resource conservation theory is a stress theory that attempts to explore the process of stress generation and the underlying mechanisms by which people cope with stressors. Over the past thirty years, this theory has been commonly used to explain the relationship between stressors and stress, and has been widely applied (Hobfoll, 1989, 2001; Hobfoll et al., 2018). As a psychological theory and organizational behavior theory, the core of this theory is to explain stressors and feelings of pressure through the flow of resources. This is very suitable for studying the problem of employee psychological resource loss caused by stress, which can well explain the sources and evolution trends of employee psychological insecurity, and explore ways to eliminate stress through reasonable allocation of resource flow. According to the theory of resource conservation, people have the motivation to establish, protect, and cultivate their own resource pool in order to protect themselves and support their social relationships. When existing resources are at risk of loss, actual losses occur, and there is no resource return after resource investment, people will experience stress reactions. The pressure that employees face comes from the internal atmosphere of the organization and its leaders. This pressure can lead to the loss of employees‘ psychological resources, thereby causing them to develop a sense of psychological insecurity. Therefore, leaders with management responsibilities need to understand the importance of employees' psychological safety and take corresponding measures.

For leaders, it is necessary to communicate with employees in daily work to release tasks, track task progress, and handle special situations. However, this process is influenced by the leader's thinking, experience, and even bias, and the output process is a one-way output from the leader to the employee. This means that in the process of communication and leadership, leaders‘ perception and thinking will follow a self-centered approach. Therefore, it is difficult for leaders to approach and solve problems from the objective situation of work tasks in a decentralized way that combines the perspectives of others. Accordingly, when leaders focus on the work itself and communicate and understand employees' psychology and needs through a decentralized way of thinking, they can significantly improve employees‘ work experience (Gao and Liu, 2021). However, most leaders find it difficult to meet these requirements in complex work. Therefore, multiple companies including Google are committed to promoting mindfulness training (attention, awareness, acceptance, communication). Those training focuses on enhancing awareness and transforming leadership models, which will help improve leaders' communication skills and problem-solving abilities (Du et al., 2023). Arendt et al. (2019) defined leader mindfulness in communication as the mindfulness trait exhibited by a leader in communicating with employees. For example, focusing on listening to subordinates‘ speeches, maintaining calmness in communication, and not making hasty judgments about what has happened. It is worth noting that mindfulness training also has some help in improving emotional intelligence. For example, mindfulness training can enhance an individual's ability to perceive emotions and help them better manage their own emotions by focusing on the present and discarding distractions. This not only benefits the upward management of leaders, but also helps them coordinate team resources and promote cross team collaboration. Existing research has shown that leader mindfulness in communication can alleviate tension within organizations and enhance employee job performance (Dane, 2011). However, the current research and discourse on the impact of leader mindfulness in communication on employee well-being and psychological capital remain insufficiently explored. In this context, this study mainly explores the impact of leader mindfulness in communication on employees' psychological safety.

The conservation of resources theory explains the psychological state of employees when faced with stress and threats, which helps us understand the relevant mechanisms of leader mindfulness in communication on employees‘ psychological safety (Hobfoll, 1989; Fatima et al., 2018). The conservation of resources theory suggests that abundant initial resources encourage individuals to fully utilize and invest in existing resources, strengthen connections between individuals, and optimize resource channels (Hobfoll, 2001; Hobfoll et al., 2018). Obviously, leader mindfulness in communication helps leaders allocate their various resources reasonably, and achieve a reasonable, balanced, and efficient solution. This means that leaders are able to uphold leader empathy when dealing with affairs and assigning tasks, taking into account the psychology, work values, and interests of employees (Dulebohn et al., 2012; Abbas et al., 2014; Deci et al., 2017). For example, leaders listen to employees' needs and provide assistance to them (Fossataro et al., 2016; Klimecki et al., 2016). And, leaders understand the value of employees‘ work through communication, thereby encouraging them to reduce career stress. Employees' psychological safety is enhanced when they perceive the goodwill of their leader's empathy. On the other hand, leader mindfulness in communication can open up resource channels and improve the competitive atmosphere, thereby freeing employees from resource constraints. Employees are more inclined to adopt a reciprocal approach to resource investment, thereby establishing an interpersonal trust (Lapidot et al., 2007; Kannan-Narasimhan and Lawrence, 2012; Saleem et al., 2020). This resource-rich environment encourages employees to engage in constructive reciprocity, thereby strengthening interpersonal trust and collective resilience.

In addition, the impact of leader empathy and interpersonal trust on employees' psychological safety is also constrained by individual level factors (Edmondson and Lei, 2014; O'Donovan and McAuliffe, 2020). In organizations, interpersonal interactions are accompanied by frequent emotional feedback (Itzchakov and Kluger, 2017; Reis et al., 2017; Weinstein et al., 2022). Therefore, feeling the kindness of others in leader empathy and interpersonal trust can have an impact on employees' psychological safety. A study by Liu et al. (2020) suggests that employee mindfulness can encourage employees to increase their work engagement and understand others' attitudes through communication and listening. A study by Wan et al. (2022) also suggests that the interactive effects between different mindfulness subjects may bring greater positive effects. Therefore, this study suggests that employees with high-level employee mindfulness can help individuals self regulate and build more positive interpersonal relationships by relying on their awareness and focus on the present moment. For example, communicating more actively and interacting more efficiently with leaders and colleagues, and deeply perceiving their help (Simione et al., 2020). It is worth noting that when both leaders and employees maintain a mindful communication and work style, it can promote closer connections among team members while maintaining a sense of unity between superiors and subordinates. Under this group consciousness, it can promote leaders and employees to work together in an open manner to address issues, create a harmonious and united work atmosphere, and enhance the role of leader empathy and interpersonal trust in employees' psychological safety. Therefore, this study examined the positive moderating effect of employee mindfulness among various variables.

**Figure 1:**
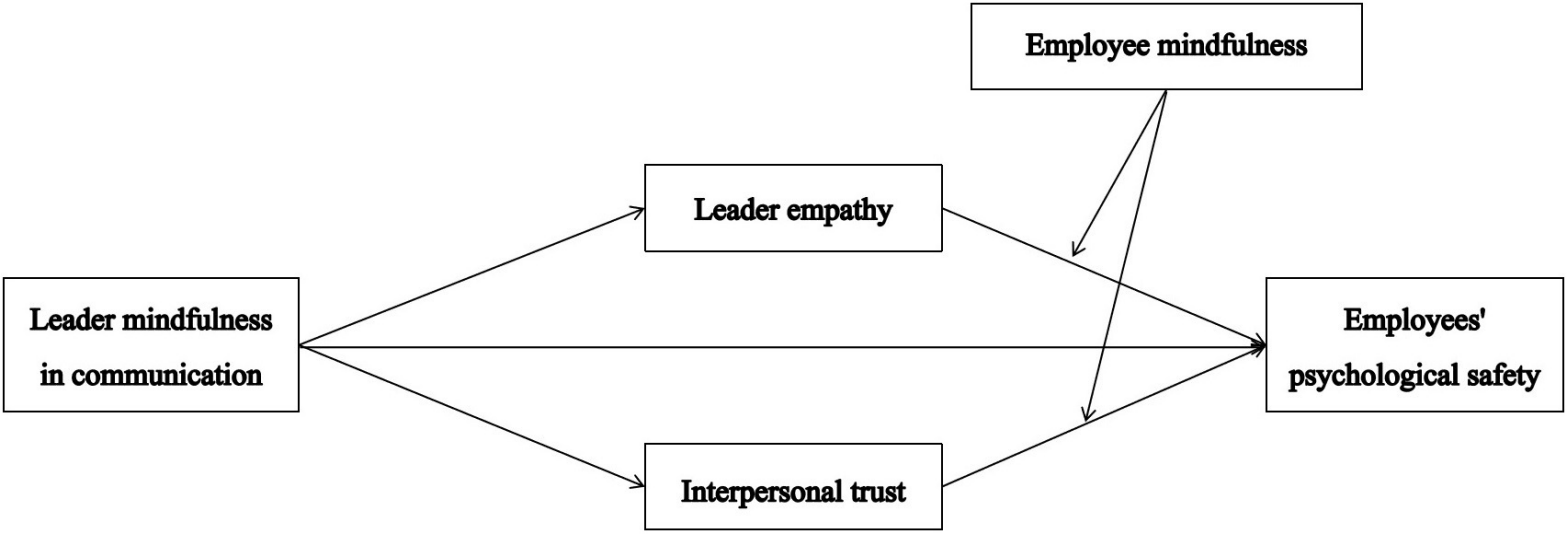
Theoretical model diagram.

Unlike previous studies that mainly focused on industrial organizations, we mainly collected samples from employees of Chinese technology companies. Chinese technology companies started late, but their growth and iteration speed is very fast, and they have gradually become an important part of China's economic growth. Faced with the ever-changing technological iteration and organizational tasks, employees of Chinese technology companies face extremely high work pressure and high personnel turnover risks, which exacerbates the psychological insecurity of technology company employees. In this atmosphere, employees‘ psychological capital issues can seriously damage the company's efficiency and employee well-being, and should be the focus of the company and leaders. In this situation, it is important for leaders to maintain a mindful communication style, empathize with employees' situations, and create a harmonious work atmosphere to alleviate their psychological problems. Our research aims to deepen our understanding of the enhancement of employees‘ psychological security and encourage business leaders to maintain mindfulness in communication with their employees. The results emphasized the importance of using mindfulness communication to promote employees' psychological security. The theoretical model diagram of this study is shown in Figure 1.

## 2 Theory and hypotheses

### 2.1 Conservation of resources theory and employees' psychological safety

Competition and pressure are everywhere in the workplace. The Conservation of Resources theory explains people's reactions to resource loss in the face of stress. This is mainly reflected in three aspects: preventing resource loss, maintaining existing resources, and actively acquiring resources (Hobfoll, 1989, 2001; Hobfoll et al., 2018). Preventing the loss of resources is crucial. Psychological resources, as one of the most important resources, are mainly reflected in employees' psychological safety, which has a decisive impact on people's behavior and attitudes (Kim et al., 2022). When the psychological safety of employees is too low, it can lead to their silent behavior and negative confrontational behavior. The Conservation of Resources theory provides the answer to this problem.

The Conservation of Resources theory suggests that employees' aversion to resource loss outweighs the pleasure of acquiring resources (Hobfoll, 2001). This means that preventing the decline of employees' psychological safety is the key to solving the problem. The relationship between employees and leaders is a work relationship, and there is a hierarchical gap (Dulebohn et al., 2012). Therefore, unless employees' upward feedback reaches a certain scale, it is difficult to arouse empathy and attention. Leaders also find it difficult to understand the stress and psychological state of their employees (Ruben and Gigliotti, 2017). This requires a leader's mindfulness in communication. Leader mindfulness in communication helps leaders maintain focus and sensitivity in their work and communication, thereby deepening their understanding of employees' psychological states and generating leader empathy. After leader empathy occurs, employees will improve their psychological safety with the help of their leaders. On the other hand, the Conservation of Resources theory suggests that resources flow within an organization, and the environment and atmosphere within the organization play a crucial role in preventing resource loss for employees (Hobfoll, 2001). Leader mindfulness in communication helps leaders to be acutely aware of disruptive people and events within the organization, and intervene accordingly (Lloyd et al., 2015). Leaders can promote the improvement of interpersonal relationships in the workplace by facilitating high-quality leader member exchange relationships. This means that organizational culture is purified, interpersonal conflicts are alleviated, thereby enhancing interpersonal trust and ultimately improving employees' psychological safety.

### 2.2 The effect of leader mindfulness in communication on employees' psychological safety

Arendt et al. (2019) defined leader mindfulness in communication as the mindfulness trait exhibited by a leader in communicating with employees. This includes multiple aspects such as focused listening, maintaining calmness, and not making hasty judgments (Good et al., 2016; Feng, 2022). This means that leaders have undergone a change in their leadership style, which includes: ① being able to achieve a more open and accepting perception; ② A more objective way of thinking, no longer centered around oneself; ③ A more objective thinking perspective that can consider issues from the perspective of stakeholders; ④ The service stance has shifted from self serving to serving others. In management practice, this will also enhance the leader's ability to solve problems, thereby making them more proactive in taking on management responsibilities. Existing research has shown that leader mindfulness in communication can promote employees to feel recognized and motivated, and is beneficial for leaders to timely understand employees‘ psychological states and needs, so as to make timely adjustments (Van Quaquebeke and Felps, 2016). For example, a study suggests that leader mindfulness in communication may have a significant impact on employee job satisfaction, job happiness, and job performance (King and Badham, 2018). A study by Daniel et al. (2020) discussed the positive impact of leader mindfulness in communication on interpersonal relationships and work attitudes. Based on these studies, our research explores the impact mechanism of leader mindfulness in communication on employees' psychological safety, in order to help leaders care for employees, promote their mental health and organizational development.

This study suggests that leader mindfulness in communication means that leaders are attentive, calm, and not judgmental, which are traits of caring for employees in the workplace. This can help leaders gain a more sensitive understanding of employees' psychological states and the problems they encounter, and intervene accordingly. Therefore, leaders tend to view problems from the perspective of their employees rather than simply conveying work pressure to them. The work pressure of employees has been alleviated, and their psychological safety has been improved. On the other hand, leader mindfulness in communication will also lead employees to feel cared for from the change in leader attitude, and believe that leaders can care about their work and psychological state (Eberth and Sedlmeier, 2012). This is beneficial for alleviating psychological problems caused by work pressure. Thus enhancing the psychological safety of employees. This study suggests that:

H1:There is a positive correlation between leader mindfulness in communication and employees' psychological safety.

### 2.3 The mediating role of leader empathy

Leader empathy is the process by which leaders perceive employees' emotions, which includes both cognitive empathy and emotional empathy (Woltin et al., 2011). Among them, cognitive empathy is an individual's tendency to understand the world from the perspective of others; Emotional empathy is the tendency of individuals to share the emotions of others. Existing research suggests that frequent and efficient communication is the foundation for achieving empathy (Preston and Hofelich, 2012). This is mainly because only through effective communication and contact can people abandon the tendency to start from themselves. Thus bringing into the perspective of others, understanding the world through their feelings, and obtaining emotional tendencies that are consistent with others. Leader mindfulness in communication encourages leaders to focus on their work and employees, and carefully listen to their ideas and opinions, providing favorable conditions for effective communication and empathy (Rahimnia and Sharifirad, 2015). Therefore, this study suggests that leader mindfulness in communication is an important antecedent to leader empathy.

In addition, a study by Batson et al. (1997) suggests that leader empathy can prompt leaders to engage in behaviors that meet employee expectations, in order to address common issues encountered by employees in the workplace. This will result in employees benefiting from leader empowerment. This study expands their research by introducing leader empathy as a mediating factor between leader mindfulness in communication and employees‘ psychological safety. Specifically, leader mindfulness in communication means high-quality communication between superiors and subordinates. Leader empathy is the result of communication, which also promotes employees' perception of the leader's care, further enhances their psychological capital, and improves their psychological safety. This study suggests that:

H2: There is a positive correlation between Leader mindfulness in communication and Leader Empathy.
H3: Leader empathy plays a mediating role between leader mindfulness in communication and employees' psychological safety.
H4: There is a positive correlation between leader empathy and employees' psychological safety.

### 2.4 The mediating role of interpersonal trust

Interpersonal trust refers to the degree to which an individual believes they can trust the language, behavior, etc. of others in their interactions with them (Lapidot et al., 2007). This is the foundation of communication and cooperation between individuals (Simons et al., 2007). Existing research suggests that fair and attentive leaders can effectively curb internal disharmony within organizations and significantly enhance interpersonal trust (Usman et al., 2021). Fairness and meticulousness are also components of mindfulness. In addition, maintaining mindfulness in communication also helps to encourage others to dare to comment on organizational matters and work tasks through reducing bias, full respect, and honest communication. Thus reducing employees' negative defensive and emotional confrontational attitudes toward leaders, and generating trust in leaders and organizations. Therefore, this study suggests that leader mindfulness in communication enables leaders to pay more attention to the present, listen more attentively to the ideas and opinions of different employees, and effectively reduce distrust behavior caused by differences in information sources. Specifically, leader mindfulness in communication will effectively enhance interpersonal trust.

In addition, existing research suggests that a harmonious organizational atmosphere is the key to maintaining a positive mindset among employees and is beneficial for enhancing their psychological capital (Bédard et al., 2014). Our research extends this theory and suggests that interpersonal trust is an important factor in enhancing employees‘ psychological safety. This is mainly because interpersonal trust helps employees reduce interpersonal stress caused by distrust and prevent others, thereby contributing to employees' psychological safety. In summary, this study suggests that:

H5: There is a positive correlation between Leader mindfulness in communication and interpersonal trust.
H6: Interpersonal trust plays a mediating role between leader mindfulness in communication and employees' psychological safety.
H7: Interpersonal trust is positively correlated with employees' psychological safety.

### 2.5 The moderating role of employee mindfulness

The Conservation of Resources theory suggests that an individual's sensitivity to resources is also influenced by their own factors (Hobfoll et al., 2018). This means that in the process of organizational resource flow, individual factors can also affect employees‘ absorption efficiency of external resources. Specifically, when leader empathy and interpersonal trust arise, high-level employee mindfulness can more accurately and sensitively perceive the goodwill and trust of leaders and colleagues, thereby activating positive emotions and engaging in high-quality interactions. The positive emotions of leaders and colleagues are important factors in promoting employees' psychological safety (Bakker et al., 2011a). Low levels of employee mindfulness can lead to a lack of focus on their current work, which can distract their energy and delay their perception. This will reduce employee communication, work quality, and hinder them from gaining positive emotions from leaders and colleagues. This is an obstacle for employees to maintain a good psychological state.

In addition, a study by Gunasekara and Zheng (2019) also explored the interactive effects between different mindfulness subjects. Individuals with high levels of mindfulness can more efficiently reinforce the positive effects caused by other mindfulness individuals. Therefore, this study takes employee mindfulness as a moderating factor to explore the driving effect of employee mindfulness on leader empathy, interpersonal trust, and employees' psychological safety. This study suggests that:

H8: Employee mindfulness plays a positive moderating role between leader empathy and employees' psychological safety.
H9: Employee mindfulness plays a positive moderating role between interpersonal trust and employees' psychological safety.

## 3 Materials and methods

### 3.1 Participants and procedures

This study invited 506 Chinese employees as survey samples to verify the relationship between leader mindfulness in communication, leader empathy, interpersonal trust, employees' psychological safety, and employee mindfulness. The author participated in the entire process of the questionnaire survey and assured the participants that the survey results will only be used for academic research. In addition, this study has explained the details of the experiment to the subjects and obtained their consent.

Due to the potential lag of leader mindfulness in communication, this study collected data twice at 4 month intervals. In the first survey, we asked 506 participants to provide demographic information and evaluated leader mindfulness in communication. Four months later, the second survey collected data on leader empathy, interpersonal trust, employees' psychological safety, and employee mindfulness. Considering factors such as resignation and job adjustment, this study received a total of 458 valid questionnaires, with an effective rate of 90.51%, which meets the requirements.

In the study sample, there were 256 males and 202 females (55.90% and 44.10%, respectively); The main age groups are 26–35 years old and 36–45 years old (36.03% and 31.22% respectively); The education level is mainly undergraduate and master's students (60.70% and 18.34% respectively); This department is mainly composed of the marketing department and the research and development department (accounting for 40.61% and 22.71% respectively).

### 3.2 Measures

All scales in this research questionnaire are composed of mature scales. Language is crucial for the accuracy of investigations. All authors were involved in the translation work. In addition, the questionnaire uses a scale of “1–5” to measure participants' opinions on the questions, with 1 indicating “strongly disagree” and 5 indicating “strongly agree”.

### 3.3 Leader mindfulness in communication

The researchers used the scale developed by Arendt et al. (2019). This scale has 9 items, such as “When I speak, my leader will listen attentively to me. The Cronbach's alpha coefficient of this scale is 0.928.

### 3.4 Leader empathy

The researchers used the scale developed by Li (2022). This scale has 16 items, such as ”My leader can accurately detect my low mood. The Cronbach's alpha coefficient of this scale is 0.956.

### 3.5 Interpersonal trust

The researchers used the scale developed by McAllister (1995). The scale has 10 items, such as “If I talk to a colleague about my difficulties, I know he/she will care about me and provide constructive feedback. The Cronbach's alpha coefficient of this scale is 0.938.

### 3.6 Employees' psychological safety

The researchers used the scale developed by (Baer and Frese (2003)). This scale has 7 items, such as ”Others won't complain about me when I make mistakes. The Cronbach's alpha coefficient of this scale is 0.915.

### 3.7 Employee mindfulness

The researchers used the scale developed by Kimmes et al. (2018). This scale has 5 items, such as “When I talk to my colleagues, we all pay attention to each other's viewpoints. The Cronbach's alpha coefficient of this scale is 0.886.

### 3.8 Control variables

The control variables for this study include age, gender, education level, and department. In addition, employees working in different departments face different job responsibilities and pressures, which may result in varying degrees of sensitivity to Employers' psychological safety.

### 3.9 Ethical approval

This study was approved by the Ethics Committee of Taizhou Polytechnic College (Codes: 20240278).

## 4 Results

### 4.1 Confirmatory factor analysis and reliability analysis

This study conducted confirmatory factor analysis using AMOS 23.0 software. The variables included include leader mindfulness in communication, leader empathy, interpersonal trust, employees' psychological safety and employee mindfulness. Table 1 shows the fit indicators of the five factor model. The fit indices of the 5-factor model (χ^2^/df = 1.187, CFI = 0.986, NFI = 0.920, TLI = 0.986, and RMSEA = 0.020) was better than other factor models. This indicates that these five variables have good discriminability and can be tested in the next step.

**Table 1:** Confirmatory factor analyses.

**Factors**	**χ^2^**	**DF**	**χ^2^/DF**	**CFI**	**NFI**	**NNFI**	**TLI**	**IFI**	**RMSEA**
Five-factor model:h	1,215.339	1,024	1.187	0.986	0.920	0.986	0.986	0.986	0.020
Four-factor model:g	3,536.646	1,028	3.440	0.821	0.766	0.812	0.812	0.822	0.073
Three-factor model:f	4,294.188	1,031	4.165	0.768	0.716	0.756	0.756	0.768	0.083
Three-factor model:e	4,810.123	1,031	4.665	0.731	0.682	0.718	0.718	0.732	0.089
Three-factor model:d	4,664.984	1,031	4.525	0.741	0.692	0.729	0.729	0.742	0.088
Two-factor model:c	5,722.257	1,033	5.539	0.666	0.622	0.651	0.651	0.667	0.100
Two-factor model:b	6,020.098	1,033	5.828	0.645	0.602	0.628	0.628	0.646	0.103
One-factor model:a	7,782.906	1,034	7.527	0.519	0.485	0.498	0.498	0.521	0.119

a: Leader mindfulness in communication + Employees' psychological safety + Leader empathy + Interpersonal trust + Employee mindfulness.

b: Leader mindfulness in communication + Employee mindfulness; Employees' psychological safety + Leader empathy + Interpersonal trust.

c: Leader mindfulness in communication + Employees' psychological safety + Employee mindfulness; Leader empathy + Interpersonal trust.

d: Leader mindfulness in communication + Employee mindfulness; Employees' psychological safety; Leader empathy + Interpersonal trust.

e: Leader mindfulness in communication + Employees' psychological safety; Leader empathy + Interpersonal trust; Employee mindfulness.

f: Leader mindfulness in communication; Employees' psychological safety + Employee mindfulness; Leader empathy + Interpersonal trust.

g: Leader mindfulness in communication; Employees' psychological safety; Employee mindfulness; Leader empathy + Interpersonal trust.

h: Leader mindfulness in communication; Employees' psychological safety; Leader empathy; Interpersonal trust; Employee mindfulness.

The average variance extraction (AVE) and composite reliability (CR) were analyzed. The AVE value measures the proportion of explanatory variance for each indicator in the construct, and the values for each variable is as follows: 0.588 for leader mindfulness in communication, and 0.576 for leader empathy, and 0.604 for interpersonal trust, and 0.607 for employees' psychological safety, and 0.608 for employee mindfulness. All values were >0.5, indicating good convergent validity.

The CR value measures the internal consistency of the construct. The CR values for each variable were as follows: 0.928 for leader mindfulness in communication, and 0.956 for leader empathy, 0.938 for interpersonal trust, and 0.915 for employees' psychological safety, and 0.886 for employee mindfulness. All values are >0.7, indicating good confidence.

### 4.2 Common method deviation test

The results of the questionnaire survey are all from employees and the data is collected in two separate sessions. The interval for collecting data is 4 months. To further validate the impact of homology errors in this study, researchers used Harman's single factor test. The results output by SPSS 22.0 software indicate that the non rotated first principal component is 34.244%, which is lower than the standard value (40%).

### 4.3 Descriptive statistical analysis and correlation analysis

The results output by SPSS 22.0 software are shown in Table 2. Table 2 shows the positive correlations among the key variables. There is a positive correlation between leader mindfulness in communication and leader empathy (*r* = 0.356, *p* < 0.01); there is a positive correlation between leader mindfulness in communication and Interpersonal trust (*r* = 0.418, *p* < 0.01); there is a positive correlation between leader ambition and employees' psychological safety (*r* = 0.451, *p* < 0.01); there is a positive correlation between interpersonal trust and employees' psychological safety (*r* = 0.486, *p* < 0.01); There is a positive correlation between leader mindfulness in communication and employees' psychological safety (*r* = 0.500, *p* < 0.01). The positive effect suggests that mindful leadership can foster a safer psychological environment for employees.

**Table 2:** Descriptive statistical analysis and correlation analysis.

**Variables**	**MEAN**	**SD**	**1**	**2**	**3**	**4**	**5**	**6**	**7**	**8**
1. Gender	1.44	0.497	1							
2. Age	2.566	1.097	0.243^**^	1						
3. Education level	3.126	0.774	0.031	−0.041	1					
4. Department	2.318	1.414	−0.162^**^	−0.114^*^	0.282^**^	1				
5. Leader mindfulness in communication	3.148	1.028	0.09	−0.047	0.091	−0.03	1			
6. Leader empathy	3.182	0.997	−0.042	−0.065	0.004	0.08	0.356^**^	1		
7. Interpersonal trust	3.198	1.055	−0.014	−0.005	0.081	0.065	0.418^**^	0.439^**^	1	
8. Employees' psychological safety	3.185	1.076	0.047	0.002	0.054	0.034	0.500^**^	0.451^**^	0.486^**^	1
9. Employee mindfulness	3.306	1.099	−0.008	−0.028	0.038	0.06	0.261^**^	0.216^**^	0.250^**^	0.493^**^

^*^p < 0.05, ^**^p < 0.01.

### 4.4 Mediating effects tests

This study referred to the methods of Preacher and Hayes (2004) and Hayes (2013) to verify the mediating effect between multiple variables. The results output by SPSS 22.0 software are shown in Table 3.

**Table 3:** Tests for intermediate effect.

**Variables**	**Dependent variable: leader empathy**	**Dependent variable: interpersonal trust**	**Dependent variable: employees' psychological safety**	
	**Model 1**	**Model 2**	**Model 3**	**Model 4**
**Control variables**				
Gender	−0.097 (−1.067)	−0.102 (−1.092)	0.024 (0.284)	0.067 (0.863)
Age	−0.024 (−0.577)	0.035 (0.847)	0.032 (0.857)	0.029 (0.854)
Education level	−0.071 (−1.212)	0.037 (0.612)	−0.007 (−0.127)	0.001 (0.013)
Department	0.061 (1.892)	0.043 (1.277)	0.022 (0.754)	−0.000 (−0.000)
**Independent variable**				
Leader mindfulness in communication	0.324^**^ (7.373)	0.396^**^ (8.756)	0.419^**^ (10.415)	0.264^**^ (6.435)
**Mediating variable**				
Leader empathy				0.217^**^ (5.147)
Interpersonal trust				0.214^**^ (5.234)
R^2^	0.156	0.204	0.392	0.484
ΔR^2^	0.145	0.193	0.384	0.475
F	F_(6, 452)_ = 13.917, *p* = 0.000	F_(6, 452)_ = 19.289, *p* = 0.000	F_(6, 452)_ = 48.627, *p* = 0.000	F_(8, 450)_ = 52.735, *p* = 0.000

^*^p < 0.05.

^**^p < 0.01.

Table 3 shows the role of mediator variables between independent and dependent variables. From Table 3, the regression coefficient of leader mindfulness in communication on employees‘ psychological safety is 0.419 (*p* < 0.01), indicating a significant positive correlation between leader mindfulness in communication and employees' psychological safety. Therefore, H1 was validated in Model 3. In Model 1 and Model 4, the regression coefficient of leader mindfulness in communication on leader endurance was 0.324 (*p* < 0.01), indicating a significant positive correlation between leader mindfulness in communication and leader endurance. The regression coefficient of leader empathy on employees‘ psychological safety is 0.217 (*p* < 0.01), indicating a significant positive correlation between leader empathy and employees' psychological safety. Therefore, H2–H4 was validated in Model 1 and Model 4. In Model 2 and Model 4, the regression coefficient of leader mindfulness in communication on interpersonal trust was 0.396 (*p* < 0.01), indicating a significant positive correlation between leader mindfulness in communication and interpersonal trust. The regression coefficient of Interpersonal Trust on employees‘ psychological safety is 0.214 (*p* < 0.01), indicating a significant positive correlation between Interpersonal Trust and employees' psychological safety. Therefore, H5–H7 was validated in Model 1 and Model 4. The positive effect indicates that leader empathy and interpersonal trust have a significant mediating effect.

### 4.5 Moderating effect analysis

This study employed the moderation effect test method, as proposed by Wen et al. (2005), to investigate the moderating influence of employee mindfulness. The results output by SPSS 22.0 software are shown in Table 4.

**Table 4:** Tests for moderating effects.

**Variables**	**Dependent variable: employees' psychological safety**					
	**Model 1**	**Model 2**	**Model 3**	**Model 4**	**Model 5**	**Model 6**
**Control variables**						
Gender	0.060 (0.717)	0.067 (0.863)	0.062 (0.816)	0.060 (0.717)	0.067 (0.863)	0.074 (0.989)
Age	0.028 (0.735)	0.029 (0.854)	0.030 (0.891)	0.028 (0.735)	0.029 (0.854)	0.029 (0.850)
Education level	−0.001 (−0.016)	0.001 (0.013)	−0.005 (−0.112)	−0.001 (−0.016)	0.001 (0.013)	−0.004 (−0.082)
**department**						
**Independent variable**						
Leader empathy	0.249^**^ (5.438)	0.217^**^ (5.147)	0.162^**^ (3.657)			
Interpersonal trust				0.260^**^ (5.859)	0.214^**^ (5.234)	0.139^**^ (3.265)
**Moderator variables**						
Employee mindfulness		0.217^**^ (5.147)	0.162^**^ (3.657)		0.325^**^ (9.292)	0.331^**^ (9.701)
**Interactive term**						
Leader empathy x employee mindfulness			0.119^**^ (3.574)			
Interpersonal trust x employee mindfulness						0.159^**^ (4.982)
R^2^	0.385	0.484	0.498	0.385	0.484	0.511
ΔR^2^	0.385	0.099	0.014	0.385	0.099	0.027
F	F_(7, 451)_ = 40.307, *p* = 0.000	F_(8, 450)_ = 52.735, *p* = 0.000	F_(9, 449)_ = 49.521, *p* = 0.000	F_(7, 451)_ = 40.307, *p* = 0.000	F_(8, 450)_ = 52.735, *p* = 0.000	F_(9, 449)_ = 52.114, *p* = 0.000

^*^p < 0.05.

^**^p < 0.01.

Table 4 substantiates the facilitative role of moderating factors in amplifying relationships among core constructs. From Table 4: ① In Model 3, the regression coefficient between the interaction term leader empathy x employee mindfulness and employees‘ psychological safety is 0.119 (*p* < 0.01), which means hypothesis H8 has been tested; ② In Model 6, the regression coefficient between the interaction term interpersonal trust x employee mindfulness and employees' psychological safety is 0.159 (*p* < 0.01), indicating that hypothesis H9 has been tested. The positive effect demonstrates a moderating role of employee mindfulness.

### 4.6 Simple efficiency analysis

**Figure 2:**
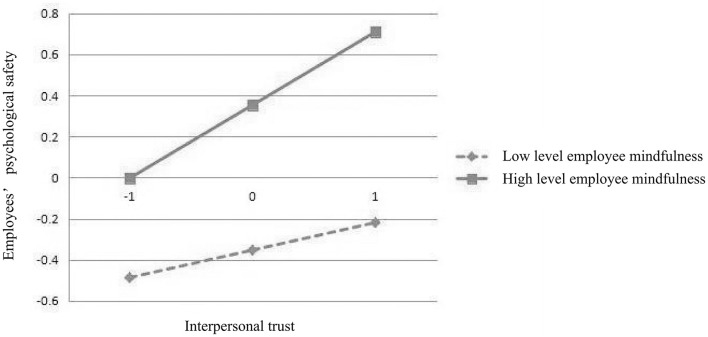
Simple efficiency analysis (H8).

**Figure 3:**
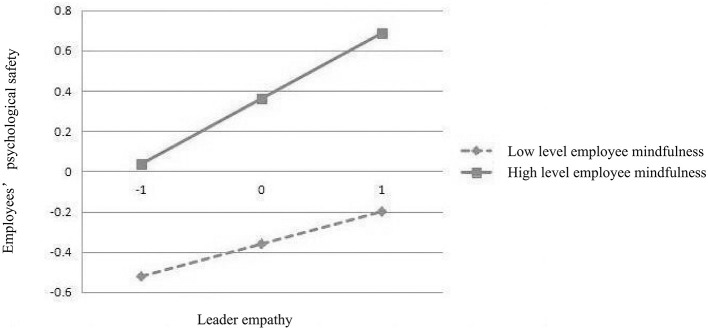
Simple efficiency analysis (H9).

In order to understand the positive regulatory effects of high-level employee mindfulness (M+1SD) and low-level employee mindfulness (M-1SD) on employees' psychological safety, this study conducted a simple slope analysis using SPSS v.22.0 software. Please refer to Figures 2, 3 and Table 5 for specific results. Figures 2, 3 show the relationship between the mediating and dependent variables caused by different levels of employee mindfulness. Table 5 demonstrates that 95% confidence intervals for effects across varying levels of employee mindfulness consistently exclude zero, indicating statistical significance at all observed mindfulness tiers.

**Table 5:** Tests for simple efficiency analysis.

**Moderating effects**	**Level**	**Regression coefficient**	**Standard error**	**95% CI**	
H8	Mean	0.093	0.025	0.052	0.147
	High level (+1SD)	0.121	0.031	0.064	0.187
	Low level (−1SD)	0.648	0.028	0.017	0.127
H9	Mean	0.108	0.025	0.064	0.164
	High level (+1SD)	0.154	0.032	0.097	0.222
	Low level (−1SD)	0.063	0.030	0.008	0.127

According to Table 5, in Simple Efficiency Analysis, the regression coefficient is a positive number and the *P*-values are all within the significant range. In addition, all 95% CIs do not include 0. The results confirm that: ① employee mindfulness plays a positive regulatory role between leader empathy and employees‘ psychological safety; ② employee mindfulness plays a positive regulatory role between interpersonal trust and employees' psychological safety.

## 5 Conclusions and discussion

### 5.1 Theoretical contributions

Firstly, this study explores the impact of leader mindfulness in communication on employees‘ psychological safety. A study by Shen et al. (2019) shows that positive leadership behavior can significantly enhance employees' psychological capital. In addition, existing research has also elucidated the role of colleagues‘ positive behavior in shaping employees' psychological security (Montani et al., 2020). Based on their work, this study has been expanded and empirical evidence has been provided. In addition, this study also demonstrated how leader mindfulness in communication can stimulate leader empathy and interpersonal trust, without bringing positive contributions to employees‘ psychological safety. This demonstrates the effectiveness of leader mindfulness in communication and the importance of employees' psychological safety.

Secondly, based on the conservation of resources theory, this study suggests that leader empathy and interpersonal trust play a mediating role in the impact of leader mindfulness in communication on employees‘ psychological safety. As a leadership style that enhances a leader's focus, leader mindfulness in communication can increase attention to employees' work progress and psychological state, thereby generating empathy for employees facing psychological resource issues and improving their psychological safety. In addition, leader mindfulness in communication can also help enhance interpersonal trust within the organization and improve employees‘ psychological safety in a trusting atmosphere. Therefore, this study elucidates the concept of leader mindfulness in communication, The impact mechanism of leader empathy, interpersonal trust, and employees' psychological safety has theoretical significance for expanding related research.

Finally, from the perspective of employees themselves, their focus and sensitivity toward work and environment can also cause psychological fluctuations. Employee mindfulness helps employees stay focused and sensitive to the present moment (Glomb et al., 2011). Therefore, this study uses employee mindfulness as a moderating variable to explore the moderating effects among multiple variables. Previous studies have lacked the interaction between different mindfulness subjects. Therefore, this study contributes to and supplements existing research, and makes a positive contribution to exploring the impact of leader mindfulness in communication on employees' psychological safety.

### 5.2 Practical implication

Our research mainly discusses the impact of leaders‘ communication styles on employees' psychological capital, as well as the positive role of mindfulness as an emerging element in leaders‘ implementation of management behavior. Our research suggests that appropriate communication between superiors and subordinates is crucial for employees' psychological security, and mindfulness helps leaders and employees improve communication efficiency and promote interpersonal trust. From an organizational perspective, it is important to focus on increasing the proportion and frequency of mindfulness training in leader and employee training, in order to provide a foundation for them to achieve organizational goals (Hülsheger et al., 2014). Overall, our research has made contributions to management practice from the following two aspects.

Firstly, this study extends psychological safety research to a fast-paced, high-pressure technology sector context in China. In China, the number of employees engaged in fast-paced and high-pressure industries is rapidly increasing. The most typical employees of this type are those in technology companies. Their psychological safety factors are being threatened by high-intensity work pressure and inappropriate leadership styles. Currently, this threat is seen as a price for obtaining high rewards, and few scholars pay attention to it. This study aims to promote mindfulness based work practices among employees and leaders in technology companies to enhance their psychological security. This will enhance the well-being of employees engaged in fast-paced and high-pressure industries.

Secondly, our research supports that organizations should widely promote mindfulness training and the application of mindfulness in communication. Especially for technology-based enterprises, they face more external opportunities, and managers and employees need to start from the work tasks themselves to improve the stability and efficiency of decision-making. In addition, employees of technology-based enterprises also face significant work pressure, requiring leaders to adopt mindfulness based communication methods to promote interpersonal trust and leader empathy, thereby enhancing employees‘ psychological safety (Jamieson and Tuckey, 2017). As a form of training that can interact with emotional intelligence, mindfulness training can enhance an individual's emotional intelligence, particularly in areas such as emotional perception, emotion regulation, and social skills. In addition, mindfulness training enhances individuals' self-awareness and helps to improve multiple dimensions of emotional intelligence. For example, mindfulness practice can improve an individual's ability to perceive and recognize their own emotional state, thereby better regulating emotional responses. Therefore, this training is crucial for both leaders and employees, as it can significantly enhance the harmonious interpersonal atmosphere within the organization and ensure the stability of the organization's human resources.

Finally, utilitarian leadership styles, especially self-centered leadership styles, are no longer applicable to the long-term development of organizations. An open, decentralized, and objective leadership style can better unite employees, shape a shared vision for the team, enhance the team's psychological capital and cohesion, and promote the well-being of the organization and employees. This is crucial for the stability of the organization's human resources. This will encourage employees to focus on the present and create a better organizational atmosphere, thereby enhancing the stability of organizational personnel and organizational performance.

## 6 Limitations and future research

There are still some limitations to this study. (1) The data of the questionnaire in this study are all from employee evaluations, so there may be a common method deviation. To avoid this factor, we have taken three screening measures. The first method is to screen two batches of data every four months. The second method is to conduct Harmer's single factor test. The third method is to check the AVE and CR of the questionnaire data. The results all prove that the error is within a reasonable range. Future research could use leader-reported measures and cross-cultural samples to validate findings. (2) At the same time, it is also possible to consider whether there are other boundary conditions that may affect the mechanism, providing guidance for promoting Employees' psychological safety.

## Data Availability

The original contributions presented in the study are included in the article/supplementary material, further inquiries can be directed to the corresponding author.

## References

[B1] Abbas M. Raja U. Darr W. Bouckenooghe D. (2014). Combined effects of perceived politics and psychological capital on job satisfaction, turnover intentions, and performance. J. Manag. 40, 1813–1830. 10.1177/0149206312455243

[B2] Arendt J. F. W. Pircher Verdorfer A. Kugler K. G. (2019). Mindfulness and leadership: communication as a behavioral correlate of leader mindfulness and its effect on follower satisfaction. Front. Psychol. 10:667. 10.3389/fpsyg.2019.00667 30984078 PMC6450257

[B3] Baer M. Frese M. (2003). Innovation is not enough: climates for initiative and psychological safety, process innovations, and firm performance. J. Organ. Behav. 24, 45–68. 10.1002/job.179

[B4] Bakker A. B. Albrecht S. L. Leiter M. P. (2011a). Key questions regarding work engagement. Eur. J. Work Organ. Psychol. 20, 4–28. 10.1080/1359432X.2010.485352

[B5] Batson C. D. Polycarpou M. P. Harmon-Jones E. Imhoff H. J. Mitchener E. C. Bednar L. L.. (1997). Empathy and attitudes: can feeling for a member of a stigmatized group improve feelings toward the group? J. Pers. Soc. Psychol. 72, 105–118. 10.1037/0022-3514.72.1.105 9008376

[B6] Bédard K. Bouffard T. Pansu P. (2014). The risks for adolescents of negatively biased self-evaluations of social competence: the mediating role of social support. J. Adolesc. 37, 787–798. 10.1016/j.adolescence.2014.05.004 25086456

[B7] Dane E. (2011). Paying attention to mindfulness and its effects on task performance in the workplace. J. Manag. 37, 997–1018. 10.1177/0149206310367948

[B8] Daniel C. Walsh I. Mesmer-Magnus J. (2020). Mindfulness: unpacking its three shades and illuminating integrative ways to understand the construct. Int. J. Manag. Rev. 24, 654–683. 10.1111/ijmr.12296

[B9] Deci E. L. Olafsen A. H. Ryan R. M. (2017). Self-determination theory in work organizations: the state of a science. Annu. Rev. Organ. Psychol. Organ. Behav. 4, 19–43. 10.1146/annurev-orgpsych-032516-113108

[B10] Du W. Yu H. Liu X. Zhou X. (2023). Mindfulness training reduces slippery slope effects in moral decision-making and moral judgment. Sci. Rep. 13:2967. 10.1038/s41598-023-29614-9 36804425 PMC9941505

[B11] Dulebohn J. H. Bommer W. H. Liden R. C. Brouer R. L. Ferris G. R. (2012). A meta-analysis of antecedents and consequences of leader-member exchange: integrating the past with an eye toward the future. J. Manag. 38, 1715–1759. 10.1177/0149206311415280

[B12] Eberth J. Sedlmeier P. (2012). The effects of mindfulness meditation: a meta-analysis. Mindfulness 3, 174–189. 10.1007/s12671-012-0101-x

[B13] Edmondson A. C. Lei Z. (2014). Psychological safety: the history, renaissance, and future of an interpersonal construct. Annu. Rev. Organ. Psychol. Organ. Behav. 1, 23–43. 10.1146/annurev-orgpsych-031413-091305

[B14] Fatima T. Majeed M. Shah S. Z. A. (2018). Jeopardies of aversive leadership: a conservation of resources theory approach. Front. Psychol. 9:1935. 10.3389/fpsyg.2018.01935 30386276 PMC6199367

[B15] Feng X. (2022). Calm down and enjoy it: influence of leader-employee mindfulness on flow experience. Psychol. Res. Behav. Manag. 15, 839–854. 10.2147/PRBM.S360880 35422665 PMC9005140

[B16] Fossataro C. Sambo C. Garbarini F. Ianneti G. D. (2016). Interpersonal interactions and empathy modulate perception of threat and defensive responses. Sci. Rep. 6:19353. 10.1038/srep19353 26839143 PMC4738254

[B17] Gao Y. Liu H. (2021). How supervisor–subordinate guanxi influence employee innovative behavior: a moderated mediation model. Psychol. Res. Behav. Manag. 14, 2001–2014. 10.2147/PRBM.S342875 34934367 PMC8684436

[B18] Glomb T. M. Duffy M. K. Bono J. E. Yang T. (2011). Mindfulness at work. Res. Pers. Hum. Resour. Manag. 30, 115–157. 10.1108/S0742-730120110000030005

[B19] Good D. J. Lyddy C. J. Glomb T. M. Bono J. E. Brown K. W. Duffy M. K.. (2016). Contemplating mindfulness at work: an integrative review. J. Manag. 42, 877–880. 10.1177/0149206315617003

[B20] Gunasekara A. Zheng C. S. (2019). Examining the effect of different facets of mindfulness on work engagement. Employ. Relat. 41, 193–208. 10.1108/ER-09-2017-0220

[B21] Hayes A. F. (2013). Introduction to Mediation, Moderation, and Conditional Process Analysis: A Regression-Based Approach. New York, NY: Guilford Press.

[B22] Hebles M. Trincado-Munoz F. Ortega K. (2022). Stress and turnover intentions within healthcare teams: the mediating role of psychological safety, and the moderating effect of COVID-19 worry and supervisor support. Front. Psychol. 12:758438. 10.3389/fpsyg.2021.758438 35173646 PMC8841584

[B23] Hobfoll S. E. (1989). Conservation of resources: a new attempt at conceptualizing stress. Am. Psychol. 44, 513–524. 10.1037/0003-066X.44.3.513 2648906

[B24] Hobfoll S. E. (2001). The influence of culture, community, and the nested-self in the stress process: advancing conservation of resources theory. Appl. Psychol. 50, 337–421. 10.1111/1464-0597.00062

[B25] Hobfoll S. E. Halbesleben J. Neveu J. P. Westman M. (2018). Conservation of resources in the organizational context: the reality of resources and their consequences. Annu. Rev. Organ. Psychol. Organ. Behav. 5, 103–128. 10.1146/annurev-orgpsych-032117-104640

[B26] Hülsheger U. R. Lang J. W. Depenbrock F. Fehrmann C. Zijlstra F. R. Alberts H. J. (2014). The power of presence: the role of mindfulness at work for daily levels and change trajectories of psychological detachment and sleep quality. J. Appl. Psychol. 99, 1113–1128. 10.1037/a0037702 25198098

[B27] Itzchakov G. Kluger A. N. (2017). Can holding a stick improve listening at work? the effect of listening circles on employees' emotions and cognitions. Eur. J. Work Organ. Psychol. 26, 663–676. 10.1080/1359432X.2017.1351429

[B28] Jamieson S. D. Tuckey M. R. (2017). Mindfulness interventions in the workplace: a critique of the current state of the literature. J. Occup. Health Psychol. 22, 180–193. 10.1037/ocp0000048 27643606

[B29] Jiang Y. Huang L. Guo Y. Yang Q. Li H. Zhou H.. (2023). The relationship between fear of COVID-19 and psychological distress in tour guides: the mediating role of job insecurity and the moderating role of psychological resilience. Psychol. Res. Behav. Manag. 16, 3107–3119. 10.2147/PRBM.S417296 37576449 PMC10423002

[B30] Kannan-Narasimhan R. Lawrence B. S. (2012). Behavioral integrity: how leader referents and trust matter to workplace outcomes. J. Bus. Ethics 111, 165–178. 10.1007/s10551-011-1199-9

[B31] Kim B.-J. Kim M.-J. Lee J. (2022). The influence of corporate social responsibility on safety behavior: the importance of psychological safety and the authentic leadership. Front. Public Health 10:1090404. 10.3389/fpubh.2022.1090404 36530700 PMC9748560

[B32] Kimmes J. G. Jaurequi M. E. May R. W. Srivastava S. Fincham F. D. (2018). Mindfulness in the context of romantic relationships: initial development and validation of the relationship mindfulness measure. J. Marital Fam. Ther. 44, 575–589. 10.1111/jmft.12296 29073322

[B33] King E. Badham R. (2018). Leadership in uncertainty: the mindfulness solution. Organ. Dyn. 48:4. 10.1016/j.orgdyn.2018.08.005

[B34] Klimecki O. Mayer S. Jusyte A. Scheeff J. Schönenberg M. (2016). Empathy promotes altruistic behavior in economic interactions. Sci. Rep. 6:31961. 10.1038/srep31961 27578563 PMC5005993

[B35] Lapidot Y. Kark R. Shamir B. (2007). The impact of situational vulnerability on the development and erosion of followers' trust in their leader. Leader. Q. 18, 16–34. 10.1016/j.leaqua.2006.11.004

[B36] Li Z. (2022). Research on the impact of leadership empathy on employee innovation performance in digital transformation: a moderated dual mediation model. Financ. Essays 2, 89–100. Available online at: https://kns.cnki.net/kcms2/article/abstract?v=hx6LgM6qJjtPJVr6VS1Yo9nRb_yRZQO4KISvCw4Px1_IVN7A8-Vj98a2Gl375Zm6GFFoKNB3ZI8al_0CYSdbRf0ZCbV2KqGWAubadw84JR2ZLctj1odXLc37DDeFMC2oBqd7c9_-IWm2-iPf6aCamXwtDcdIyFFvfjoe0f2Fgfk=&uniplatform=NZKPT

[B37] Liu C. Liu S. Yang S. Wu H. (2019). Association between transformational leadership and occupational burnout and the mediating effects of psychological empowerment in this relationship among CDC employees: a cross-sectional study. Psychol. Res. Behav. Manag. 12, 437–446. 10.2147/PRBM.S206636 31297001 PMC6598747

[B38] Liu J. Xu R. Wang Z. (2024). The effects of psychological capital, work engagement and job autonomy on job performance in platform flexible employees. Sci. Rep. 14:18434. 10.1038/s41598-024-69484-3 39117745 PMC11310468

[B39] Liu S. Xin H. Shen L. He J. Liu J. (2020). The influence of individual and team mindfulness on work engagement. Front. Psychol. 10:2928. 10.3389/fpsyg.2019.02928 32038356 PMC6985205

[B40] Lloyd K. J. Boer D. Keller J. W. Voelpel S. (2015). Is my boss really listening to me? the impact of perceived supervisor listening on emotional exhaustion, turnover intention, and organizational citizenship behavior. J. Bus. Ethics 130, 509–524. 10.1007/s10551-014-2242-4

[B41] McAllister D. J. (1995). Affect- and cognition-based trust as foundations for interpersonal cooperation in organizations. Acad. Manag. J. 38, 24–59. 10.2307/256727

[B42] Montani F. Vandenberghe C. Khedhaouria A. Courcy F. (2020). Examining the inverted U-shaped relationship between workload and innovative work behavior: the role of work engagement and mindfulness. Hum. Relat. 73, 59–93. 10.1177/0018726718819055

[B43] Obrenovic B. Jianguo D. Khudaykulov A. Khan M. A. S. (2020). Work-family conflict impact on psychological safety and psychological well-being: a job performance model. Front. Psychol. 11:475. 10.3389/fpsyg.2020.00475 32296367 PMC7137557

[B44] O'Donovan R. McAuliffe E. (2020). A systematic review exploring the content and outcomes of interventions to improve psychological safety, speaking up and voice behaviour. BMC Health Serv. Res. 20:101. 10.1186/s12913-020-4931-2 32041595 PMC7011517

[B45] Preacher K. J. Hayes A. F. (2004). SPSS and SAS procedures for estimating indirect effects in simple mediation models. Behav. Res. Methods Instruments Comput. 36, 717–731. 10.3758/BF03206553 15641418

[B46] Preston S. D. Hofelich A. J. (2012). The many faces of empathy: parsing empathic phenomena through a proximate, dynamic-systems view of representing the other in the self. Emot. Rev. 4, 24–33. 10.1177/1754073911421378

[B47] Rafiei S. Souri S. Nejatifar Z. Amerzadeh M. (2024). The moderating role of self-efficacy in the relationship between occupational stress and mental health issues among nurses. Sci. Rep. 14:15913. 10.1038/s41598-024-66357-7 38987325 PMC11237126

[B48] Rahimnia F. Sharifirad M. S. (2015). Authentic leadership and employee well-being: the mediating role of attachment insecurity. J. Bus. Ethics 132, 363–377. 10.1007/s10551-014-2318-1

[B49] Reis H. T. Lemay E. P. Finkenauer C. (2017). Toward understanding understanding: the importance of feeling understood in relationships. Soc. Pers. Psychol. Compass 11:e12308. 10.1111/spc3.12308

[B50] Ruben B. D. Gigliotti R. A. (2017). Communication: sine qua non of organizational leadership. Theory Pract. 54, 12–30. 10.1177/2329488416675447

[B51] Saleem F. Zhang Y. Z. Gopinath C. Adeel A. (2020). Impact of servant leadership on performance: the mediating role of affective and cognitive trust. SAGE Open 10:2158244019900562. 10.1177/2158244019900562

[B52] Shen C. Yang Y. He P. Wu Y. J. (2019). How does abusive supervision restrict employees' feedback-seeking behavior? J. Manag. Psychol. 34, 546–559. 10.1108/JMP-10-2018-0480

[B53] Simione L. Raffone A. Mirolli M. (2020). Stress as the missing link between mindfulness, sleep quality, and well-being: a cross-sectional study. Mindfulness 11, 439–451. 10.1007/s12671-019-01255-y

[B54] Simons T. Friedman R. Liu L. A. McLean Parks J. (2007). Racial differences in sensitivity to behavioral integrity: attitudinal consequences, in-group effects, and “trickle down” among Black and non-Black employees. J. Appl. Psychol. 92, 650–665. 10.1037/0021-9010.92.3.650 17484548

[B55] Usman M. Ghani U. Cheng J. Farid T. Iqbal S. (2021). Does participative leadership matter in employees' outcomes during COVID-19? Role of leader behavioral integrity. Front. Psychol. 12:646442. 10.3389/fpsyg.2021.646442 34093327 PMC8177206

[B56] Van Quaquebeke N. Felps W. (2016). Respectful inquiry: a motivational account of leading through asking open questions and listening. Acad. Manag. Rev. 43, 5–27. 10.5465/amr.2014.0537

[B57] Wan J. Liu Z. Zhang X. Liu X. (2022). Congruence in leaders-subordinates' mindfulness and knowledge hiding: the role of emotional exhaustion and gender similarity. Front. Psychol. 13:1007190. 10.3389/fpsyg.2022.1007190 36389527 PMC9650406

[B58] Weinstein N. Itzchakov G. Legate N. (2022). The motivational value of listening during intimate and difficult conversations. Soc. Pers. Psychol. Compass 16:e12651. 10.1111/spc3.12651

[B59] Wen Z. L. Hou J. T. Zhang L. (2005). Comparison and application of moderating effect and mediating effect. Acta Psychol. Sin. 37, 268–274. Available online at: https://kns.cnki.net/kcms2/article/abstract?v=hx6LgM6qJjvglehrpN76uuWb63zhr1_HR_mbmr5sEHOUdUue9elxRqFicgiSUJiHEdfnBY9SrRqi5zpTueLEMDIP87-1Wwup1oJVoSC7ONunpVcEGJ4iInOCDkmgl8dUZdxJ6rhZ3JwoQns2ofEfnPYb7MMW2Zzd&uniplatform=NZKPT

[B60] Woltin K.-A. Corneille O. Yzerbyt V. Y. Förster J. (2011). Narrowing down to open up for other people's concerns: empathic concern can be enhanced by inducing detailed processing. J. Exp. Soc. Psychol. 47, 418–427. 10.1016/j.jesp.2010.11.006

